# A survey of the level of knowledge and understanding of members of the inpatient team on the role of the physician associate on the general adult psychiatric wards

**DOI:** 10.1192/bjo.2021.398

**Published:** 2021-06-18

**Authors:** Declan Hyland, Mohammed Uddin

**Affiliations:** 1 Consultant Psychiatrist, Clock View Hospital, Liverpool, Mersey Care NHS Foundation Trust; 2 Physician Associate, Clock View Hospital, Liverpool, Mersey Care NHS Foundation Trust

## Abstract

**Aims:**

Physician Associates (PAs) are healthcare professionals with a general medical education background, having completed a two-year postgraduate degree. Whilst the number of PAs employed in healthcare trusts continues to increase, the number working in mental health settings remains small.

Mersey Care NHS Foundation Trust employed two PAs two years ago. In August 2019, a third PA was recruited to work at Clock View Hospital, a general adult inpatient unit.

This survey aims to establish what level of understanding different members of the inpatient teams across the inpatient wards have of the tasks PAs are permitted to undertake and those they are not.

**Method:**

A survey was designed, listing 37 tasks, e.g. completing an admission clerking. For each task, the participant was asked whether a PA is allowed to complete it or not, with three options provided – “can carry out the task”, “cannot carry out the task” and “do not know.” A score of + 1 was awarded if the correct answer was provided, –1 for an incorrect answer and 0 if the respondent didn't know. The highest possible score for a completed survey was + 37 points; the lowest possible score was –37 points.

A sample of survey respondents was identified from the three general adult inpatient wards at Clock View Hospital and the Psychiatric Intensive Care Unit (PICU), comprising: senior doctors, junior trainees, Ward Manager, Deputy Ward Manager, Band 5 nurse and Assistant Practitioner.

**Result:**

Twenty-four members of staff completed the survey – 3 senior doctors, 4 junior trainees, 4 Ward Managers, 4 Deputy Ward Managers, 5 Band 5 nurses and 4 Assistant Practitioners. The respondents were distributed equally across the three general adult wards and the PICU. The highest survey score was 36 out of 37 (a Consultant); the lowest was 18 (a junior trainee). The lowest mean score was variable across the different grades of staff, with Consultants scoring highest at 29 and Assistant Practitioners and Ward Managers both scoring lowest at 25. There was little variability in mean score (only 2 points) across the three wards and PICU.

**Conclusion:**

The results from this survey demonstrate that different members of the inpatient team have a good understanding of what tasks PAs are and are not permitted to. There is still a need to provide further education to inpatient staff to ensure they utilise the PA at Clock View Hospital appropriately and that the PA is able to develop his skill set.

